# Establishing advanced practice for medical imaging in New Zealand

**DOI:** 10.1002/jmrs.44

**Published:** 2014-02-25

**Authors:** Jill Yielder, Adrienne Young, Shelley Park, Karen Coleman

**Affiliations:** 1 University of AucklandAuckland, New Zealand; 3 University of OtagoWellington, New Zealand

**Keywords:** Advanced practice, career progression, role development, role profiles

## Abstract

**Introduction**This article presents the outcome and recommendations following the second stage of a role development project conducted on behalf of the New Zealand Institute of Medical Radiation Technology (NZIMRT). The study sought to support the development of profiles and criteria that may be used to formulate Advanced Scopes of Practice for the profession. It commenced in 2011, following on from initial research that occurred between 2005 and 2008 investigating role development and a possible career structure for medical radiation technologists (MRTs) in New Zealand (NZ).

**Methods**The study sought to support the development of profiles and criteria that could be used to develop Advanced Scopes of Practice for the profession through inviting 12 specialist medical imaging groups in NZ to participate in a survey.

**Results**Findings showed strong agreement on potential profiles and on generic criteria within them; however, there was less agreement on specific skills criteria within specialist areas.

**Conclusions**The authors recommend that one Advanced Scope of Practice be developed for Medical Imaging, with the establishment of generic and specialist criteria. Systems for approval of the overall criteria package for any individual Advanced Practitioner (AP) profile, audit and continuing professional development requirements need to be established by the Medical Radiation Technologists Board (MRTB) to meet the local needs of clinical departments. It is further recommended that the NZIMRT and MRTB promote and support the need for an AP pathway for medical imaging in NZ.

## Introduction

In 2005, the New Zealand Institute of Medical Radiation Technology (NZIMRT) began an investigation into role development for Medical Radiation Technologists (MRTs) through a 3-year research project that aimed to investigate the need for advanced practice roles in both medical imaging and radiation therapy, and to recommend a possible structure for career progression. The investigation was predicated on the growing body of knowledge, arising particularly from the United Kingdom (UK), that demonstrates the capability of MRTs to extend their role into non-traditional areas, performing these to high levels of expertise when they have undertaken appropriate postgraduate education and experience.^1^ It confirmed the perception that New Zealand (NZ) MRTs wish to obtain clinical advancement through an extended career progression framework, as opposed to advancement through management roles, and believe that this would increase job satisfaction, recruitment and retention for the profession. Several studies were performed within this project that demonstrated that NZ MRTs are capable of performing extended roles.^1^ The study added local evidence to two decades of research evidence and supported role development as an important part of the evolution of the profession.

The research project culminated in a recommendation that the profession introduce a three level career framework consisting of Assistant Practitioner, Practitioner and Advanced Practitioner (AP), with the emphasis being placed initially on the development of an AP pathway for medical imaging and radiation therapy.^1^ These three levels were conceptualised as being similar to the first three levels of career progression in the UK, with the difference between Practitioner and AP focusing on clinical leadership (see general criteria in Table 1) and the development of role extension activities appropriate to the specialist area.^2^ This recommendation was accepted, initiating a second stage of research, which was commenced in 2011. This stage sought to support the development of profiles and criteria that may be used to formulate Advanced Scopes of Practice for the profession. This article reports on the results of the medical imaging aspects of this research. A separate companion article will report the results from radiation therapy.^3^

**Table 1:** Key generic criteria.

Generic criterion	Frequency	% Agreement
Lead role in planning and delivering high-quality clinical practice	87	91
Advanced knowledge of the specified area	89	93
Liaison with the multi-disciplinary team	81	84
Prioritising and decision-making*	81	84
Leading improvements and advances	85	89
Interpretation of quality assurance and feedback for best practice; overview of quality assurance	84	88
Ongoing education of staff and students, leadership, research, evidence-based practice	88	92
Providing support and advice to the patient and family*	65	68

* These criteria were not presented in the Research/Education profile.

## Methodology

An online survey, utilising the software ‘Survey Monkey™ (Armonk, NY), was used to distribute and collect a questionnaire electronically. A snowball sampling technique (non-probability sampling that utilises participants to recruit further participants who they know to have the appropriate expertise) was used to elicit participants from several sources across 12 specialist areas of medical imaging practice. Senior MRTs in the full range of modalities being investigated were initially identified by a review of the Medical Radiation Technologists Board (MRTB) register and a link to the online survey was sent directly to those whose contact details were known to the researchers or indirectly via the MRTB. The survey link was also forwarded to representatives of three hospital departments who had been involved in previous exploratory working groups with a request to distribute to all interested staff members. A further request was sent by email to a range of radiology managers from District Health Boards (DHBs) and private radiology practices requesting them to either send the researchers the contact details of interested, suitable participants, or to send the questionnaire link directly to interested parties. Finally, an open invitation was made at the annual NZIMRT conference to any further interested parties, particularly those who were involved in clinical education, an area in which there had been few respondents at that time. The survey was open for 8 weeks from mid-March to mid-May 2012, then for a further 2 weeks following the NZIMRT conference. In total, 96 responses were received. Nine of the responses were incomplete, however, the data that were provided was of sufficient quality to be included in the analysis. The size of the senior MRT population is not known and cannot be ascertained from the public register. Of the 2299 MRTs currently registered to practice, if we were to predict that the most experienced MRTs across specialist areas would represent 10% of the total diagnostic MRT population, then respondents would represent ∼42% of the study population.

Ethics approval was obtained from The University of Auckland Human Participants Ethics Committee on 28th October, 2011 for a period of 3 years.

## Results

### Participant characteristics

**Figure 1:**
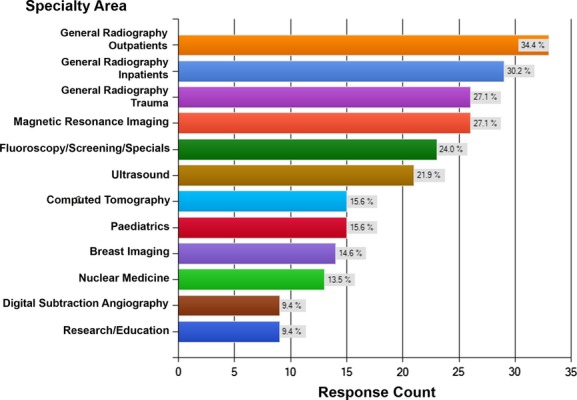
Specialty area of participants

The participants were predominantly aged 30–59 (81/95; 85.3%) and female (79/95; 83.2%). The majority of the participants work in public radiology departments (63/94; 67%) although private radiology practices were also well represented (26/94; 27.7%). A small number of respondents indicated that they worked at a tertiary education institute (5/94; 5.3%). The size of the department was relatively evenly spread across small (29/93; 31.2%), medium (33/93; 35.5%) and large (31/93; 33.3%) departments. The number of years practicing since qualification ranged from 4 to 40 years (average = 23 years). The number of respondents working in each of the 12 profiles presented ranged from 9 to 33 (average = 19) (see Fig. 1). Twenty-seven per cent (26/94) had previous experience working in an extended role, the majority of which had taken place in NZ (23/26; 88%) and consisted of reporting (16/26; 61.5%) or IV cannulation and/or drug administration (6/26; 23.1%). The remaining participants with extended role experience had performed this in the UK. Thirteen of the 16 participants (81%) who indicated that they had reporting experience identified as working in ultrasound.

### Endorsement of profiles and criteria

Due to the extensive results generated in this section across the 12 specialist areas, only the key criteria that were identified as generically important for AP roles, along with one example of specialist-specific results are presented. Table 1 illustrates the high level of agreement with the generic criteria.

**Figure 2:**
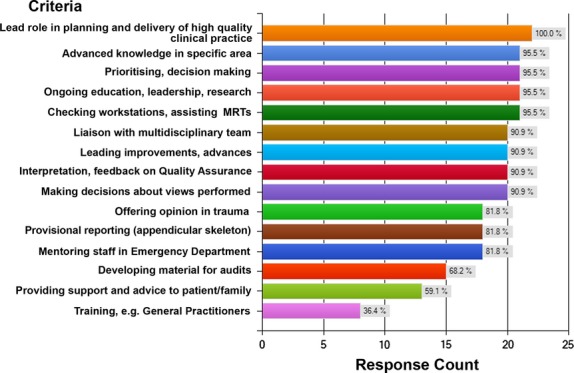
General Trauma profile

Specific clinical skills within the profiles reached variable levels of agreement. Figure 2 illustrates an example of the criteria and degrees of agreement established for the General Trauma profile.

### Open-ended question results

The third section of the questionnaire presented three open-ended questions to elicit open response. Results are presented thematically in order of decreasing frequency, with numbers indicated in brackets.

1. Are there any potential advanced practice profiles missing? Please identify them.
  Four additional profiles were suggested as possibilities for inclusion: Theatre (4); Cardiac imaging (3); Intensive care (2); Academic educator (2).
2. Please comment on any strengths or limitations within your departments that may impact on the future development of Advanced Practice roles.
  Fifty-eight participants identified strengths and/or limitations within their department that may impact on the future development of Advanced Practice roles. Refer to Table 2 for departmental strengths and limitations.
3. Do you foresee any barriers to implementing any of the advanced practice roles mentioned above in NZ? If so, please identify them.
  There were 63 respondents to this question. Refer to Table 3 for a list of perceived barriers.

**Table 2:** Perceived department strengths or limitations impacting on advanced practice roles.

Question 2 comments	Frequency	Percentage
Departmental strengths		
Workplace support	8	14
MRT enthusiasm	6	10
Radiologist support	3	5
Multidisciplinary approach	2	3
Radiologist shortage	1	2
Departmental limitations		
Lack of radiologist support	20	34
Money	17	29
Shortage of time for MRTs	14	24
Small size of NZ	8	14
Change in established practices/MRT apathy	6	10
Supporting education/training deficiencies	4	7
Believe they are already performing AP role	3	5
Legal/regulatory restrictions	3	5
Nursing APs	2	3

MRT, medical radiation technologist; AP, advanced practitioner.

**Table 3:** Perceived barriers to advanced practice implementation in New Zealand.

Question 3 comments	Frequency	Percentage
Radiologist resistance	40	63
Lack of resources/management support	14	22
Financial compensation/recognition	13	20
Small population size	9	14
MRT resistance to further education/apathy	7	11
Regulatory requirements	6	9
Referrer/patient non-acceptance	4	6
Structured, standardised framework	3	5
Legal issues	3	5
Vested interests/politics	3	5
Nurse resistance	2	3

MRT, medical radiation technologist.

## Discussion

### Profiles

Twelve AP profiles were presented relating to the range of different medical imaging sub-specialties shown in Figure 1. The only comment specifically regarding the proposed profiles was related to general radiography being separated into three different areas. Several respondents believed that these should be combined, with comments such as:

As a general MRT I think it would be hard to do either inpatient or outpatient or trauma because all MRTs work in all areas in our department. (R16)

### Criteria

A variety of criteria were suggested for each of the 12 profiles, the first eight criteria being generic across all profiles (excluding Research/Education where only 6/8 of these criteria were presented – See Table 1). For seven of eight of these key criteria, the average rate of agreement was between 84% and 93%. However, the eighth criterion (providing support and advice to the patient and family) received significantly less support with only 68% of participants in agreement. Specific comment on exclusion of this criterion was extensive and across all profiles, with many MRTs adamant that this should remain the role of other professionals:

Providing support and advice – care needed here; traditionally it is left to the GP or clinic to relay diagnosis and future planning to patient – to avoid conflicting information. (General, outpatient MRT, R21)

A possible contributor to this opinion may be that the curriculum for undergraduate medical imaging programmes in NZ has traditionally been based on a technical rationality model, emphasising the physical as opposed to social sciences. Providing advice and support for patients has not been part of the curriculum and the respondents clearly felt outside their comfort zone when considering this more personal function that stresses relationship rather than technicality.

The remaining clinical skills criteria suggested within each profile varied in number and type, depending on the specific modality or area of practice. There were no suggestions for addition or removal for any of the profiles. Only 15 of the 167 proposed criteria had an agreement of less than 50%. Twenty-three reached an agreement of 100%.

Overall, the most common reasons for excluding suggested criteria across the profiles were: the opinion that they were another professional's role; lack of knowledge; time constraints; lack of support from other professions; the perception that the criterion was already part of standard practice; or that they were role extension activities rather than Advanced Practice criteria. Specifically, the following criteria had less than 20% support: ‘joint injections pre-CT’ (3/21; 14.3%); ‘renal services fluoroscopy for example, Tenckoff placements’ (4/25; 16%); ‘joint injections under fluoroscopy’ (3/25; 12%); and ‘joint injections pre-Magnetic resonance imaging (MRI)’ (5/26; 19.2%). For a variety of reasons, there is significant reluctance by MRTs to perform interventional procedures such as joint injections, with specific comments such as:

Joint injections pre CT – see this more as radiologists role? You would need to be doing it a lot in your department to get confident and competent at this. Feel it would depend on your CT department and the volumes of these procedures. (CT-MRT, R62)

A common reason for criteria exclusion across a number of profiles was that respondents thought that some of the presented criteria described current expected practice of senior/charge MRTs. For example:

Dose reduction, post processing, prioritising, decision-making, MDT liaison are something all CT MRT's should do. (CT MRT, R33)

This highlights a general lack of understanding about Advanced Practice roles, assuming that *only* APs perform extended tasks, and that role extension is synonymous to Advanced Practice. While the more complex procedural and cognitive aspects of an AP's role can only be undertaken with appropriate education and clinical experience, there will be many extended role activities carried out by other staff working in the area who are deemed competent to undertake those tasks. The AP provides the leadership, development and assurance of best practice for these activities. A further misunderstanding within the modalities of MRI, nuclear medicine and ultrasound related to the impression that the postgraduate qualification required for registration means that everyone in those modalities already operates at AP level. This is not the case, although pre-registration education and training may enhance the capability for the practitioner to work at an advanced level in the future and to elicit support from radiologists.

The data indicate that it is important that care is taken to develop key competencies for APs that are focused on generic leadership attributes, rather than role extension activities. Generalisation of criteria rather than definition of precise tasks is important, as it is likely that these roles will be developed within specific workplaces with particular needs. Furthermore, role extension activities often become part of routine duties over time, as demonstrated by the evolution of IV cannulation into the MRI technologist role.^4^ As this occurs, it is likely that new activities will evolve. It will be the AP's role to ascertain the evidence base for these activities, lead their introduction into practice, teach staff and students and to evaluate their effectiveness in terms of quality of patient diagnosis and care, and department efficiency. As such, AP roles must remain sufficiently flexible to be future-proofed.

### Strengths and limitations

The strengths identified within the participants' current context included workplace support, for example, strong “support for change (that comes) from working for a progressive company” (R18). One participant commented that: “Our department would strongly encourage this and already financially supports the radiographers' ongoing education” (R12). A cautionary comment, however, reminds us that:

You would need forward thinking and supportive managers who would ensure that a project like this could go ahead. A change in management or finances for a DHB (District Health Board) could see the whole thing sidelined or inhibited (R49).

The enthusiasm of MRTs was also identified as a strong point, with some making reference to the forward thinking members of their departments being ready to embrace this role, and that: “There is overwhelming support for the development of this role on part of MRTs” (R61). Some also think that there is now support from radiologists, for example: “The willingness and desire of radiologists to promote and support the development of such roles within NZ” (R38). One participant commented on a shortage of radiologists in this context:

I certainly think that the smaller district hospitals have a real need for APs within Radiology. These are the places that have trouble attracting radiologists but that are still required to provide a quality service to their patients (R84).

Conversely, and consistent with previous research in NZ,^1^ the largest perceived barrier to Advanced Practice was lack of radiologist support:

MRTs are discouraged from asking questions of radiologists, and communication is channelled through senior staff MRTs… While this type of dissociation persists, I believe it will prove difficult for MRTs to have their worth and ability recognised by the radiologists who could best support and benefit from them (R43).

Although a number of years have passed since the survey of NZ radiologists was undertaken that demonstrated a moderate level of support, particularly from those who had worked with APs overseas,^1^ the perception of radiologist resistance remains. Importantly, a radiologist shortage was one of the key factors that enabled the establishment of Advanced Practice roles in the UK,^5^ therefore, areas in NZ where there is no shortage of radiologists may not provide the necessary training and ongoing support for MRTs pursuing AP positions.

It would be interesting to establish whether radiologist resistance is a reality, or whether it is a perception formed from an enduring cultural environment of medical dominance, bought into by a degree of apathy, or learned helplessness, on the part of MRTs. This may prevent them from fully recognising and exercising their potential.^6^,^7^ Apathy was raised as a limitation by a number of participants, for example:

In reality, MRTs are actually in my opinion better skilled and positioned to take on some of the role extension that nurses have already taken on. The real issue is the general apathy on the MRT's part (R10).

It appears to take the form of general apathy, not wanting to undertake further study, or a reluctance to “step on other professions' toes” (R87). This may prove a difficult barrier to overcome, even although over the past decade Advanced Practice roles in the UK have become firmly embedded, with a 2008 survey conducted by the University of Hertfordshire (cited in^8^) showing radiographers to be reporting in 20 separate areas of practice. This kind of development has created the opportunity to extend, upskill and raise the profile of the entire profession.

The second largest identified barrier to Advanced Practice was significant concern regarding the level of managerial support, including the need for appropriate financial support and remuneration for the roles, as also identified in the first phase of role development research.^1^ Some participants thought that DHBs would welcome AP development, as they have in other professions such as nursing, provided it does not cost them anything. The participants were clear that MRTs pursuing higher levels of responsibility would expect a higher salary:

Unless there are financial gains to be made, there may be limited interest in pursuing the role. In the public system there may be difficulty in obtaining a higher salary to recognize the role (R15).

A lack of understanding by management or a lack of clarified roles and responsibilities for the AP position were identified as potential reasons for a lack of financial support for such roles.

While there are fiscal constraints in the current environment, it could be important to view the establishment of AP roles as an impetus to reconceptualise how departments have traditionally been organised. If, as claimed, APs help to increase workflow, create efficiencies and decrease waiting lists (see for example^9^), then the appointment of an AP to lead an area may actually save money, particularly since that role will include ensuring best practice through leadership, trial, implementation and evaluation of new developments. One participant drew attention to the level of frustration experienced by MRTs when they are not able to have productive input into the organisation and operation of the department:

Currently the mindset of the practice is for MRTs to get on with performing imaging with limited or no input into policies being created. This is a constant of frustration, particularly when some of the ideas proposed will likely improve productivity and job satisfaction (R56).

The other limitations cited related to the shortage of time MRTs would have to pursue an Advanced Practice role, the small size of many departments, and the MRT population of NZ. With respect to the former, several comments related to departments being short staffed and unable to release MRTs to attend training for these roles, for example: “High demand within ever increasing busy practices can restrict time to develop and implement these roles amongst staff” (R25). Coupled with this is the perception that the small size of many departments would mean that specialist AP roles would not be viable – in these centres a broad role would need to be developed. Furthermore, due to the narrow and/or high workloads of many departments that would restrict the availability of appropriate positions, it was envisaged that there would be only a limited number of both AP positions and suitable candidates available.

Medico-legal aspects relating to AP roles were raised by only two participants as a limitation, with the comment made that:

Most senior staff welcome the recognition and introduction of APs but are reluctant to make a stand always stating that their arms are tied by red tape i.e., the MRT Board or the official Radiologist Board (R44).

**Figure 3:**
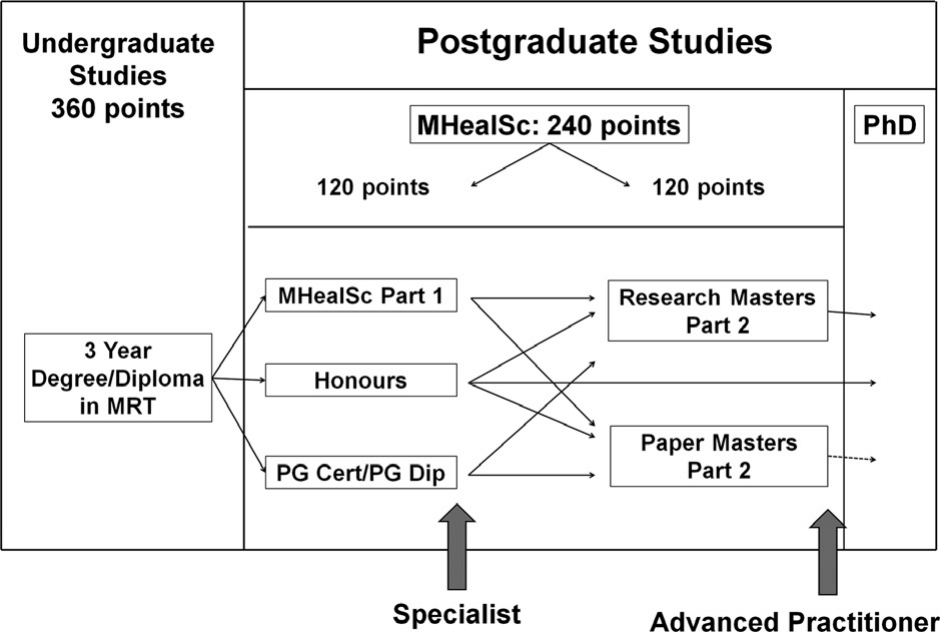
Potential education framework for extended roles and advanced practice^3^,^12^

As stated by Yielder et al. 1 all health professional groups are legally responsible for their own actions, therefore the development of the MRT role into areas of Advanced Practice will require regulation through the MRTB, with clear criteria that are regularly audited to ensure that standards of practice are upheld. Where a radiologist delegates tasks to suitably trained MRTs, they are still responsible for supervising those delegated tasks, which means that they retain ultimate responsibility for the management of the patient.^10^ The onus, therefore, is on the MRT (and MRTB) to ensure suitable education and training. The minimum education standard for AP roles would need to be at Masters level, along with evidence of clinical competence in extended role activities, to ensure international credibility.^1^,^11^ However, it may be useful for departments to utilise the Clinical Specialist level existing within some employment contracts to recognise those MRTs undertaking extended role activities who do not wish to take on the overall leadership required for AP roles (See Fig. 3). The profession would need to support NZ education providers in developing appropriate courses to enable sufficient numbers for viability.

Two participants identified that currently one limitation is the lack of academic courses to support these roles, particularly given the difficulty of setting up courses with limited numbers. Careful planning will be necessary with a focus on generic elements and, initially, a limited number of specialist skills. Education institutions will need MRTs to indicate that they are ready for this development, regardless of whether they are assured financial and status rewards in the future, before investing in course implementation.

A number of participants acknowledged workplace support and it may be that these departments are the leaders in supporting the development of the first APs in NZ. Ideally, a department that has the tripartite support of managers, radiologists and MRTs will be the front runners in this stage of national professional development.

…we are considering this – and have been for a while – it needs just a couple of people to have the courage and enthusiasm to get it started, so that others can see it's place in the NZ Health system – as a diagnostic (and therapeutic) tool MI will be relied on more and more into the future. The way we do things must change to cope with these future demands – there needs to be a paradigm shift not only in radiology but in health as a whole (R42).

While this study has provided data that meet the purpose of seeking to develop the profiles and criteria for AP profiles in NZ, it needs to be noted that a limitation of the survey is that response rates cannot be identified when using a snowball sampling technique. However, since the purpose of the research was to use senior MRTs across specialties to develop profiles that will be subject to review and further refinement, the researchers maintain that this form of purposive sampling has more validity than a random sampling of MRTs. The largest population of MRTs registered in NZ is located in general imaging (76%) and hence a random sampling would not be representative of the specialist areas.

## Conclusions

The research results indicate that in NZ, the small population of MRTs (2299 currently registered to practice) and diversity of provision in radiology departments means that separate Advanced Scopes of Practice for different specialist areas is unlikely to be sustainable. Instead, given the high degree of agreement for the generic criteria, it would suggest that the way forward lies with one Advanced Scope of Practice for each of Medical Imaging and Radiation Therapy, with common generic criteria to be included for all APs, and specialist criteria (from a suggested list) to be negotiated depending on department need. The profile development could be guided by the AP capabilities outlined in the College of radiographers' documentation on Advanced Practice and the Curriculum Framework^2^,^13^ in the UK. The MRTB would need to develop a system for the approval of the overall profile of generic plus specialist criteria for each individual AP profile application to meet Advanced Scope of Practice requirements. This process would also need to include ongoing audit to ensure standards of practice, and specific continuing professional development requirements for APs.

From these results it can be seen that although there is a strong agreement on potential profiles and the generic criteria that could lie within them, there are still barriers that may impede the implementation of Advanced Practice roles. Some of these relate to the culture of subservience and apathy that has characterised the profession since the 1940s,^6^,^7^,^14^ others relate to the level of management, radiologist and financial support that would be required. While radiographers in the UK have made extraordinary headway in the past two decades to reclaim their expertise, there still remains a marked gap between what is occurring in NZ and Australia, and the UK, that limits our international recognition and ability to attract and maintain high quality staff.^15^ The response to both phases of role development research in NZ indicates that it is imperative that an Advanced Scope of Practice is available for those MRTs and departments that wish to champion the development of the profession into an era that maintains international credibility.

### Recommendations

There are several recommendations that emerge from this research. These have been crafted jointly with the companion radiation therapy article, as it is important that career development is considered as an integrated and consistent model for the whole profession. The authors recommend that:

- The NZIMRT and MRTB promote and support the development of an AP pathway for medical imaging in NZ.
- There is one advanced scope of practice for the future career pathway, titled AP, with generic and specialised criteria for each accepted profile.
- A Masters degree is the educational requirement for an AP role
- A postgraduate diploma is the educational requirement for specialist roles; for practitioners undertaking extended role activities but not in a formalised AP position.
- The MRTB develops appropriate standards of practice and specific continuing professional development requirements for the AP role.
- The University of Auckland (as the only provider of postgraduate medical imaging programmes in NZ) works with clinical medical imaging departments to identify service needs for AP roles.
- Funding is identified to support the education and training requirements for each AP role.
